# Protective Role of Oleuropein against Acute Deltamethrin-Induced Neurotoxicity in Rat Brain

**DOI:** 10.7508/ibj.2015.04.009

**Published:** 2015-10

**Authors:** Ali Reza Khalatbary, Elmira Ghaffari, Behrooz Mohammadnegad

**Affiliations:** 1 *Molecular and Cell Biology Research Center, Dept. of Anatomy, Faculty of Medicine, Mazandaran University of Medical Sciences, Sari, Iran; *; 2 *Dept. of Anatomy, Faculty of Medicine, Mazandaran University of Medical Sciences, Sari, Iran*

**Keywords:** Deltamethrin, Oleuropein, Apoptosis

## Abstract

**Background:**

Deltamethrin (DM) is a synthetic pyrethroid insecticide that can elicit neurotoxicity, leading to apoptosis. There is accumulating evidence that oleuropein (OE) has anti-apoptotic effect. The purpose of this study was to determine the anti-apoptotic effect of OE pretreatment in the neuronal cells of cerebral cortex.

**Methods:**

Rats were randomly divided into four groups each containing five rats: DM-treated group (12.5 mg/kg, a single dose), OE-treated group (20 mg/kg per day), DM + OE-treated group, and vehicle group. Sections of the brain were obtained 24 hours after DM injection and studied for histopathological and immunohistochemistry assessment.

**Results:**

The histopathological assessments showed lesser characteristics of neural degeneration in DM + OE group compared with DM group. Greater Bcl-2 and attenuated Bax expression could be detected in the DM + OE treated-mice compared with DM group.

**Conclusion:**

The results suggested that DM-induced neurotoxicity can be subsided by OE.

## INTRODUCTION

Deltamethrin (DM) is a type II synthetic pyrethroid insecticide used as a major class of insecticides in agriculture worldwide [1]. Acute exposure to DM can elicit neurotoxicity and can be characterized by ataxia, loss of coordination, hyperexcitation, convulsions, and paralysis [2]. Neurotoxicity of DM is mediated by a series of cellular, molecular, and biochemical cascades, including the modification of sodium channels kinetics [3], increasing neurotransmitter release [4], S100β upregulation [5], induction of oxidative damage [6], and induction of cytochrome P450s [7]. Moreover, *in vitro* and *in vivo* studies have suggested the important role of apoptosis in neurotoxicity of DM [8, 9]. Apoptosis or programmed cell death is a key mechanism of neurodegenerative diseases, which is triggered by toxins, radiation, hypoxia, oxidative stress, ischemia/reperfusion, loss of survival/trophic factors, and DNA damage [10]. A number of studies have revealed that exposure to DM significantly affects the survival of neurons in rat brain and induces mitochondria-mediated apoptosis [11, 12]. Each treatment, which interrupts the apoptosis processes, could improve the DM neurotoxicity. Within the previous decades, a rapidly growing number of natural polyphenol compounds have been described to have anti-apoptotic effects. One of the main sources of these molecules is olive oil. Olive oil is a rich source of polyphenolic components similar to its main component oleuropein (OE, 3, 4 dihydroxy-phenylelenolic acid), which have many beneficial health effects on human [13-15]. There is accumulating evidence that has attributed the beneficial effects of OE and its derivatives to a variety of biological activities, including free radical scavenging/antioxidant actions, anti-inflammatory effects, and anti-carcinogenic as well as anti-apoptotic properties [15, 16]. In this regared, some experimental studies have documented that OE and its derivatives have anti-apoptotic effects on intestinal ischemia/reperfusion injury [17], 6-hydroxydopamine-induced PC12 cell apoptosis [18], and doxorubicin-induced cardiomyopathy [19]. 

Accordingly, in this work, we evaluated the effect of OE on the activity and the expression of apoptotic criteria against acute DM-induced neurotoxicity in rat brain.

## MATERIALS AND METHODS

***Animals.*** Female adult Sprague–Dawley rats (180–200 g, Pasteur Institute, Tehran, Iran) were used in this study. The animals were kept under standard conditions and fed a standard rat chow and drinking water *ad libitum* throughout the study period. DN and OE were purchased from Sigma (Germany).The rats were randomly allocated into four groups, each containing 5 rats: (1) DM-treated group (a single intraperitoneal dose of 12.5 mg/kg) [12]; (2) OE-treated group (intraperitoneally at a dose of 20 mg/kg per day for 7 days) [20]; (3) DM + OE-treated group was given pretreatment of OE for 7 days at 20 mg/kg per day with a single intraperitoneal dose of 12.5 mg/kg DM on the seventh day; (4) vehicle group. 

***Histopathological assessment.*** Brain samples were obtained 24 hours after DM injection, fixed in 10% (wt./vol.) PBS-buffered formaldehyde and embedded in paraffin. The coronal sections (5 µm) of frontal cortex were selected randomly using a microtome. For histopathological assessment, some tissue sections were deparaffinized with xylene, stained with hematoxylin-eosin (H & E) and cresyl violet, and studied using light microscopy (DME; Leica Microsystems Inc., Buffalo, NY, USA). All the histological studies were performed in a blinded fashion.

***Immunohistochemistry*** ***.*** For immunohistochemistry, the sections of frontal cortex were incubated in the goat serum (in order to block nonspecific site), polyclonal rabbit anti-Bax antibody (1:50 in PBS, vol./vol., Abcam, USA), or anti-Bcl-2 rabbit polyclonal antibody (1:100 in PBS, vol./vol., Abcam, USA) at 4°C overnight. The sections were then washed with PBS and incubated with secondary antibody conjugated with horseradish peroxidase (goat anti-rabbit IgG, Abcam, USA) for 2 hours and detected by diaminobenzidine tetrahydrochloride for 5 minutes. Afterwards, they were dehydrated and mounted. For negative controls, primary antibodies were omitted. For quantitative analysis, immunohistochemical photographs (5 photos from each samples collected from all rats in each experimental group) were assessed by densitometry using MacBiophotonics Image J 1.41a software on an ASUS personal computer. 

***Statistical analysis.*** Statistical analysis was carried out using the SPSS package (version 15, Chicago, IL, USA)., and the results were presented as mean values (±SD). The K-S test was used to evaluate the normality of the data. Also, the Tukey׳s multiple comparison test and the analysis of the variance were used to compare each of the two groups as well as compare the data among the groups, respectively. A value of *P* < 0.05 was considered statistically significant.

## RESULTS

**Fig. 1:**
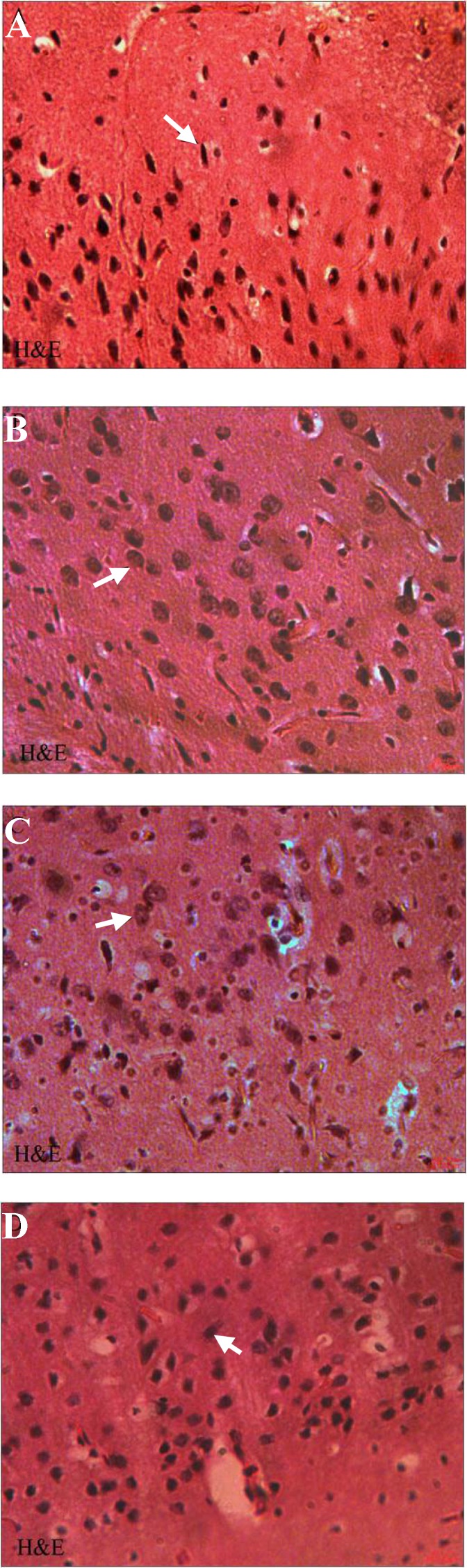
Hematoxylin-eosin staining of paraffin sections from the brain of DM (A), OE (B), DM + OE (C), and vehicle (D) treated rats. Many neuronal cells of cerebral cortex showed characteristics of degeneration with pyknosis of nuclei and shrinkage of cytoplasm in DM group , ×400. Little or no signs of degeneration were seen in OE, DM + OE, and vehicle groups, ×400. Arrows show DM and vehicle groups

**Fig. 2:**
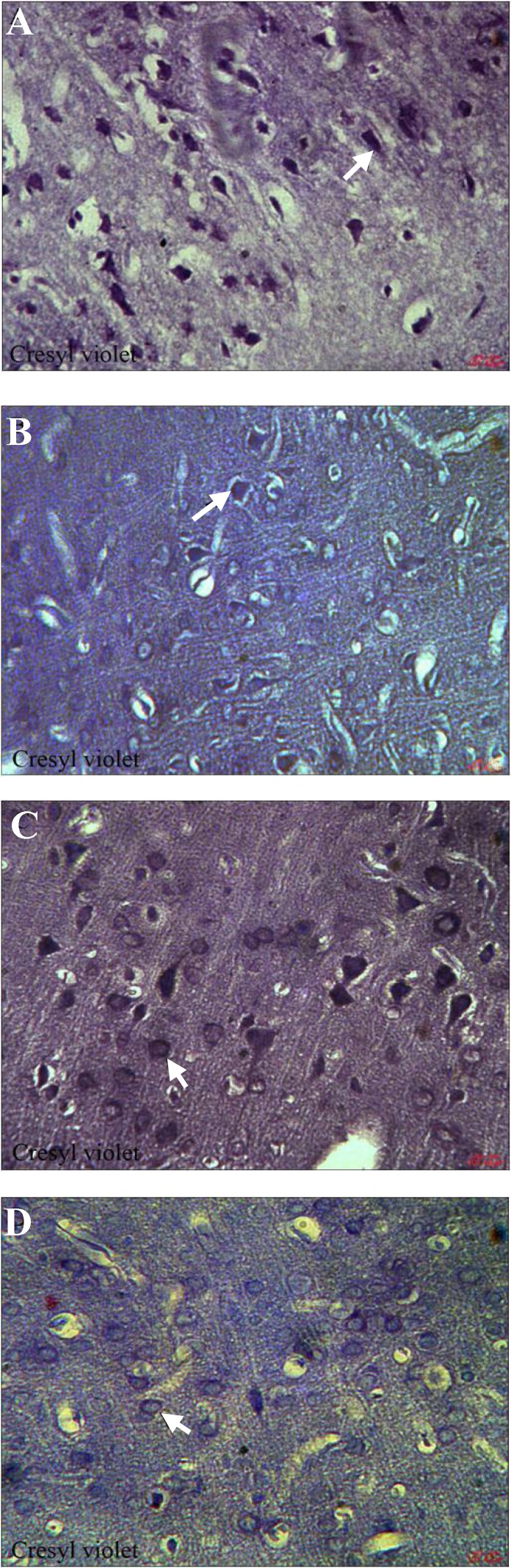
Cresyl violet staining of paraffin sections from the brain of DM (A), OE (B), DM + OE (C), and vehicle (D) treated rats. Many neuronal cells of cerebral cortex showed characteristics of degeneration with shrinkage and strong staining of Nissl bodies in DM group, ×400. Little or no signs of degeneration were seen in OE, DM + OE, and vehicle groups, ×400. Arrows show DM and vehicle groups

***Histopathological assessments.*** To observe the morphological characteristics of cortical neurons in rat brain of all experimental groups, the H & E and cresyl violet staining were used in the present study. Histopathological study with H & E staining showed that some degenerative changes in cortical neurons (pyknosis of nuclei and shrinkage of cytoplasm) (Fig. 1A), and with cresyl violet staining, it indicated shrinkage and strong staining of Nissl bodies in the brain of DM-treated rats (Fig. 2A). However, little or no signs of degeneration were seen in OE-treated (Fig. 1B and 2B) and DM + OE-treated groups (Fig. 1C and 2C) or in the vehicle group (Fig. 1D and 2D).

**Fig. 3:**
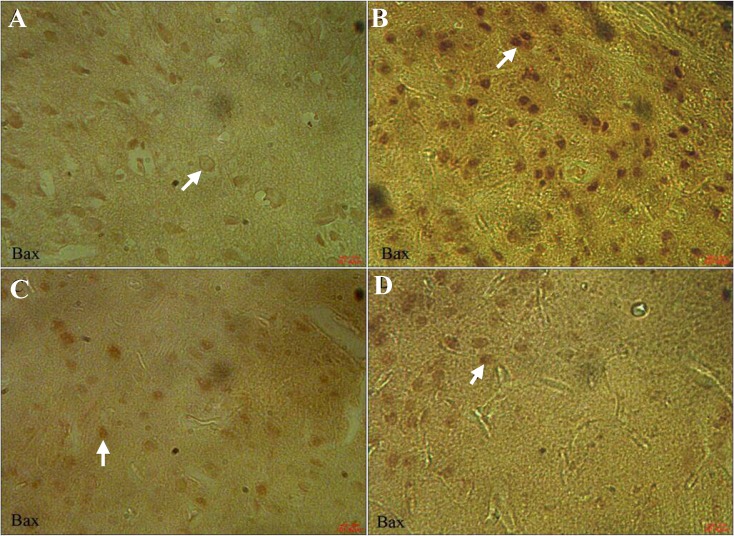
Light photomicrographs showing immunohistochemical expression of Bax in OE (A), DM (B), DM + OE (C), and vehicle (D) treated groups (magnification × 400). The positive staining of Bax is presented by the brown color of cytoplasm (arrows

**Fig. 4:**
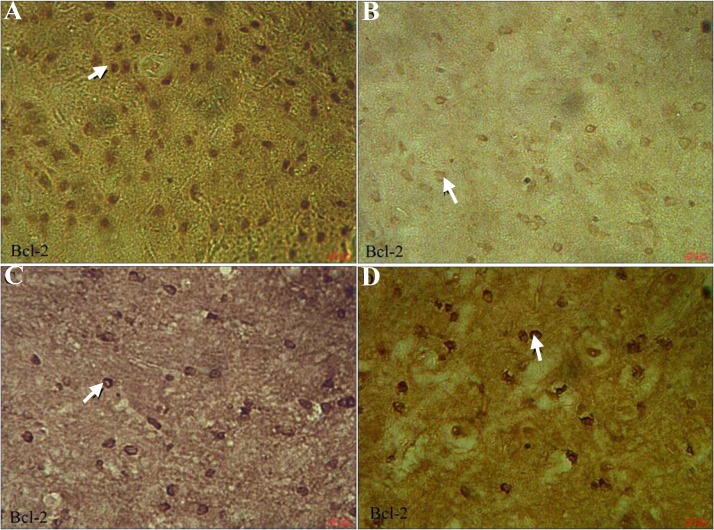
Light photomicrographs show immunohistochemical expression of Bcl-2 in OE (A), DM (B), DM + OE (C), and vehicle (D) treated groups (magnification × 400). The positive staining of Bcl-2 is presented by the brown color of cytoplasm (arrows).

***Immunohistochemistry.*** Figure 3 shows the immunohistochemical staining of Bax in all groups. Cortical neurons of the brain from OE (0.71 ± 0.05) (Fig. 3A) and vehicle-treated rats (0.63 ± 0.03) (Fig. 3D) indicated a weak positive immunoreaction for Bax, whereas the sections of DM-treated rats exhibited a strong positive staining for Bax (9.64 ± 2.19) (Fig. 3B). OE treatment in DM + OE-treated rats reduced the degree of positive staining for Bax (1.64 ± 0.28) (Fig. 3C). Figure 4 shows the immunohistochemical staining of Bcl-2 in all groups. The expression of Bcl-2 was strong in cortical neurons of the brain from the OE- (9.52 ± 1.93) (Fig. 4A) and vehicle-treated rats (9.59 ± 2.20) (Fig. 4D). In contrast, it was weak in the DM-treated rats (0.83 ± 0.11) (Fig. 4B) compared to the up-regulation in the DM + OE-treated rats (5.12 ± 0.80) (Fig. 4C). 

**Fig. 5:**
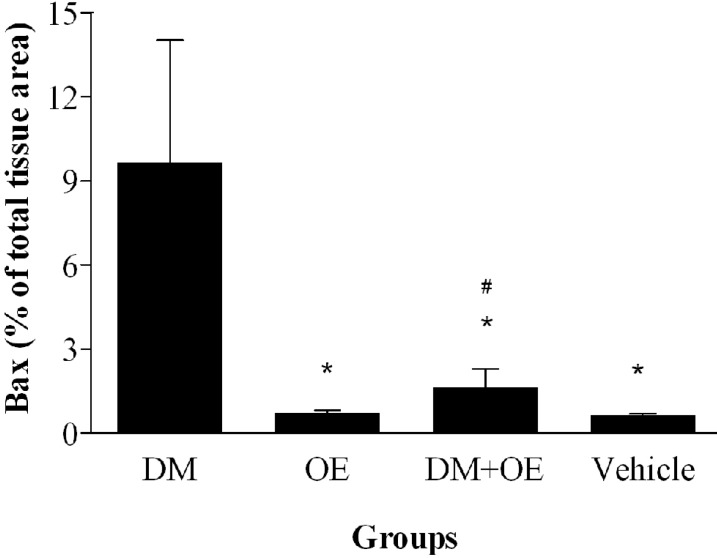
Densitometry analysis of immunohistochemical photomicrographs for Bax. Data are expressed as the percentage of total tissue area. *P < 0.001 versus DM group; ^#^*P* > 0.05 versus OE and vehicle groups. Bars indicate the standard deviations of the mean (SEM)

**Fig. 6:**
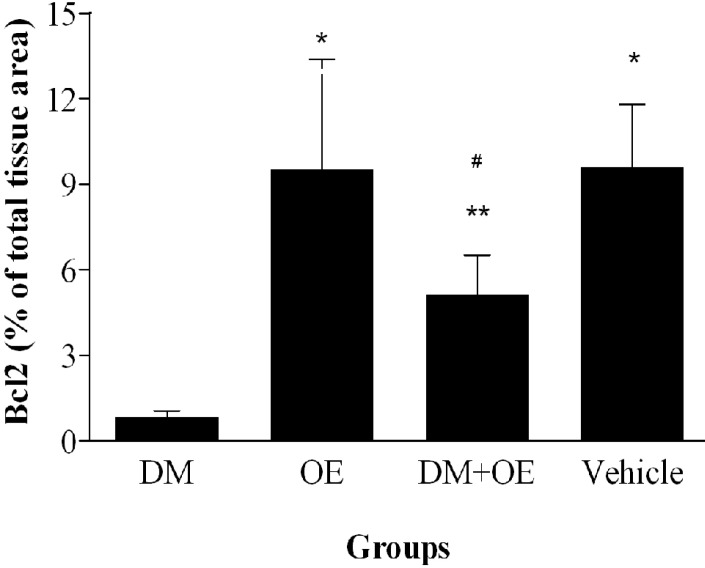
Densitometry analysis of immunohistochemical photomicrographs for Bcl-2. Data are expressed as a percentage of total tissue area. *P < 0.01 versus DM group; **P > 0.05 versus DM group; ^#^*P* > 0.05 versus OE and vehicle groups. Bars indicate the standard deviations of the mean (SEM

***Quantitative analysis.*** The histograms of the quantitative analysis of Bax and Bcl-2 staining in the experimental groups are shown in Figures 5 and 6, respectively.

## Discussion

Neurotoxins are well known risk factors for chronic neurodegenerative diseases. Although molecular mechanisms involved in the pathogenesis of iseases remain unclear, oxidative stress, excitotoxicity, inflammation, and apoptosis have been implicated as possible causes on neurodegener-ation [21]. Apoptosis is a key molecular mechanism of neurodegenerative diseases that is regulated by the Bcl-2 family proteins [21]. Among these proteins, Bcl-2 and Bax play anti-apoptotic and pro-apoptotic roles, respectively [22]. The ratio of Bax to Bcl-2 determines the cell fate; excess Bcl-2 leads to the survival of cells, while Bax induces apoptosis [23, 24]. *In vitro* and *in vivo* studies have shown that apoptosis is a key mechanism of DM neurotoxicity that is mediated by altered expression of P53, Bax, Bcl-2, and caspases [9, 13, 25]. Caspase is a family of cysteine proteases that play essential roles in apoptosis neurodegenerative [26]. P53 is a tumor suppressor gene that can activate or repress transcription as well as induce apoptosis [27]. Chen *et al.* [11] demonstrated that DM may have an effect on mitochondria-mediated apoptosis of nerve cells in rat brain by altered expression of cytochrome *c*. The cytochrome complex is a small heme protein, which is involved in initiation of apoptosis [28]. DM causes apoptosis through its interaction with Na+ channels, leading to calcium overload and activation of the ER stress pathway [9]. Results of our immune-histochemical assessment showed that the treatment with DM increased positive staining for Bax, whereas exhibited a decreased positive staining for Bcl-2 in the neuronal cells of cerebral cortex of DM group. To date, the majority of epidemiological studies involving olive oil is linked to a decreased incidence of certain types of neurodegenerative diseases such as Alzheimer’s [29], multiple sclerosis [30], and aging [31]. Animal and human studies have demonstrated that olive oil phenolic compounds are highly bioavailable. In this regard, a recent study has shown that after a single ingestion of olive oil phenolic compounds, they were absorbed, metabolized and distributed through the blood stream to practically all parts of the body of rat, even across the blood-brain barrier [32]. On the other hand, *in vitro* studies have suggested that anti-apoptotic properties of OE and its derivatives are potential neuroprotective mechanims against neurodegenerative diseases [33]. Results of our immunohistochemical assessment showed that the treatment with OE reduced positive staining for Bax, while on the contrary, it increased positive staining for Bcl-2 in the DM + OE-treated group, thereby provided the molecular evidence for the neuroprotective activity of OE. In this regard, González-Correa *et al.* [34] documented that lactate dehydrogenase efflux, as a marker of brain cell death, inhibited in a concentration-dependent manner after 7 days of oral treatment with hydroxytyrosol in rat brain slices subjected to hypoxia-reoxygenation. An* in vitro* study has also indicated that the olive oil phenolic extract and one of its constituents, gallic acid, which exerts anti-apoptotic effect against H_2_O_2_-induced apoptotic cell death in Hela cells with reduction of time-dependent caspase 9 activity [35]. Furthermore, another study documented that the incubation of PC12 cells with OE could decrease cell damage and reduce biochemical markers of apoptotic cell death including activated caspase 3, Bax/Bcl-2 ratio, and DNA fragmentation in 6-hydroxydopamine-induced PC12 cell apoptosis [19]. Histological and molecular examinations demonstrated that OE aglycone modulated apoptosis pathway, as shown by tunel staining and Bax/Bcl-2 expressions in a murine model of intestinal ischemia/reperfusion injury [18]. A recent study has shown that OE prevents doxorubicin-induced cardiomyopathy through the modulation of kinases such as Akt [20], a serine/threonine-specific protein kinase that plays a key role in apoptosis and cell proliferation [36]. 

In the present study, it is clear that DM exposure resulted in alternations of Bax/Bcl-2 expressions and apoptosis in the neuronal cells of cerebral cortex, while OE pre-exposure provided protection against DM-induced apoptosis in terms of histopathological and immunohistochemical expression of the pro- and anti-apoptotic protein. In conclusion, this study suggests that OE has modulatory effects on DM-induced apoptosis in the neuronal cells of rat cerebral cortex.
